# Verifying Sequences that Enhance Splicing

**DOI:** 10.1371/journal.pbio.0020323

**Published:** 2004-08-31

Identifying the causative mutation for a disease can be the first step to a potential cure. This task is not always trivial. Often the initial strategy is to look for the variations within a mutated gene that alter its protein coding sequence, as these mutations often alter the gene's function. However, in a growing number of cases, the causative mutation is a “synonymous” mutation—a change in the coding sequence of a gene that doesn't change the sequence of the protein coded by the gene. This type of mutation may be responsible for Seckel syndrome, a human disease characterized by dwarfism. In Seckel syndrome, the mutation doesn't alter the protein sequence itself but instead results in the skipping over of a portion of the protein coding sequence (an exon), a process called altered splicing. The disease-causing potential of this type of splicing mutation has only recently gathered attention.

Splicing assembles the exons of a transcribed gene (the RNA copy) into the right order while removing the non-coding sequences of the RNA (introns). This highly regulated process is coordinated by a number of sequences within a gene, including splice sites that precede and follow the exon, as well as by exonic splicing enhancers (ESEs), which help recruit the factors (proteins) necessary to insure proper splicing. Although splice sites have optimal (consensus) sequences, there is some variability amongst individual splice site sequences that allows splicing to take place to a greater or lesser extent. ESEs facilitate splicing, especially when a gene's splice sites vary from the consensus sequence. Candidate ESEs have previously been identified based on their more frequent occurrence in exons that are adjacent to non-consensus splice sites.

In this issue of *PLoS Biology*, William Fairbrother et al. investigated the functionality of these putative ESEs. If they are functional, the authors reasoned, then mutations that disrupt them would be selected against—that is, these mutations would tend to be discarded—in the human genome.

To this end Fairbrother et al. developed a computational method, which they call VERIFY (for “variant elimination reinforces functionality”), to evaluate the selective pressure on ESEs. They took advantage of a public database of all single nucleotide polymorphisms (DNA changes at a single point) within the human genome and compared them to the chimpanzee genome; this allowed the authors to infer the identity of the ancestral gene (or allele). By determining which allele is ancestral and which is the variant, the researchers could then distinguish the mutations that created ESEs from mutations that disrupted ESEs.

Mutations that altered or disrupted ESEs were under-represented, leading the authors to conclude that predicted ESE sequences evolve under a more stringent level of selection than exonic sequences with no predicted ESEs. This selective pressure was greater for predicted ESEs located near the splice signals than for ESEs that were located within the exon. This result was consistent with experimental findings that ESE strength diminishes with distance from the splice site.

As more vertebrate genomes are sequenced and the public database of single nucleotide polymorphisms continues to grow, this type of computational method will become increasingly valuable. It can help confirm the functionality or role of candidate regulatory elements thought to control various aspects of gene expression, and in so doing, offer insights into the complex machinations required to maintain the healthy operation of the human genome.

